# Annotations

**Published:** 1907-02-23

**Authors:** 


### ANNOTATIONS.

### Nocturnal and Diurnal Sleep.

It is generally recognised that sleep in the daytime is not so refreshing or recuperative as the customary sleep at night. Those who are compelled by reason of their work to reverse their waking and sleeping hours, frequently suffer in health from an inability to obtain the proper length and soundness of sleep during the daytime. It is a question how far this is due to custom or whether there are in addition other factors involved. M. N. Vaschide has made some observations on forty-one subjects, twenty of whom were compelled to work at night and sleep in the day. He finds that sleep in the day is more superficial, less continuous, and less recuperative than night sleep. All the functions of the body—heart, heat, respiratory movements—which are diminished automatically during nocturnal sleep, are liable to disturbances and variations in rate and rhythm in diurnal sleep. Sleep in the day does not reach the depth of nocturnal sleep, except in cases of extreme mental or physical fatigue. The pupil, which is usually contracted in normal deep sleep, is less so in deep sleep during daytime, and dilatation of the pupil excited experimentally is not so evident nor so rapid. Custom only slowly increases the duration of diurnal sleep, but this is especially influenced by complete darkness and silence. As one would naturally suppose, M. Vaschide is led to conclude that there is a close relationship between the darkness of the night and the depth and automatic characteristics of the nocturnal sleep.

### The Principalship of Glasgow University.

It will not be questioned that the University of Glasgow has gained, by the appointment of Dr. Donald MacAlister, a highly distinguished and capable Principal. Equally it may be said that the new Principal will find in the University an opportunity worthy of his great abilities. The problems which, under existing conditions, are offered by the Scottish Universities are both numerous and varied, and their solution is likely to demand wise and far-seeing statesmanship. It cannot be questioned that the changes introduced by the last Scottish Universities Commission have had many beneficial effects. At the same time, experience has shown that some modifications are in the highest degree advisable. Indeed, at the present time there is a state of unrest and dissatisfaction which will hardly be allayed except by legislative interference. This in its turn may mean that once again the Universities are to be thrown into the melting-pot, with possibilities that their best friends hardly care to contemplate. In addition to intramural and inter-University questions, there is the position created by the establishment of the Carnegie Trust, which involves many delicate questions of finance. Thus the University of Glasgow may well be considered fortunate in being able, amidst the present and impending difficulties, to count on the guidance of so experienced a man of affairs as Principal MacAlister. It is not necessary in a medical journal to describe the new Principal's attainments or to remind our readers of the evidence he has given that scholarship does not necessarily exclude business efficiency. Probably the majority of our readers, while not grudging the special claim Glasgow will now have on Dr. MacAlister, will be particularly interested to know that this claim will not prevent the Principal from continuing to act as President of the General Medical Council. There he has already accomplished most excellent work, and the Council has learned from experience to rely on his sane and steady guidance.

### Medical Inspection of Schools in Japan.

Recent events have compelled the whole civilised world to give attention to the achievements, and also to the plans and prospects, of the wonderful island-nation which has almost suddenly leapt into fame. The naval, military, and political organisations of Japan have been the texts for many writers, and have excited widespread wonder and admiration. There has now appeared a careful and exhaustive study of the educational methods favoured by this extraordinary people, and it will, we venture to say, produce an equally stirring sensation. The work has been undertaken by Mr. W. A. Sharp, Professor of Philosophy in the Elphinstone College, Bombay, and it is based on a personal investigation of some 150 institutions and on numerous interviews with individuals of all possible ranks and ages. Considering recent discussions in this country, it is hardly consoling to our national vanity to find that Japan has had a system of medical inspection of schools since 1888. Every child on entering school has to be medically examined, and this examination is repeated at intervals, the medical attendant visiting the school periodically for this purpose. Nor is this all. Arrangements are made for the occasional discussion of questions of personal and domestic hygiene, and these discussions sometimes include the parents as well as the children. The teachers are also expected to take a personal interest in the health of the pupils under their care, and to make regular inquiries on this point. A set of simple rules on the care of the health is given to every child, and these have to be learned and practised. Again, physical training is now compulsory in Japanese education. It includes gymnastics, fencing, wrestling, outdoor sports, and long walks, and, together with these, opportunity is taken to inculcate the moral worth and dignity of such qualities as chivalry, courtesy, unselfishness, and courage. It would seem that in the educational world, as in so many other directions, Japan is, to put it mildly, not a whit behind the Western nations.

